# What Do Eye Gaze Metrics Tell Us about Motor Imagery?

**DOI:** 10.1371/journal.pone.0143831

**Published:** 2015-11-25

**Authors:** Elodie Poiroux, Christine Cavaro-Ménard, Stéphanie Leruez, Jean Michel Lemée, Isabelle Richard, Mickael Dinomais

**Affiliations:** 1 LUNAM, Université d’Angers, Laboratoire Angevin de Recherche en Ingénierie des Systèmes (LARIS), EA 7315 F-49000, Angers, France; 2 LUNAM, Université d’Angers, Département de Médecine Physique et de Réadaptation, CHU d’Angers, 4 rue Larrey, 49933, Angers, Cedex 9, France; 3 LUNAM, Université d’Angers, Département d’Ophtalmologie, CHU d’Angers, 4 rue Larrey, 49933, Angers, Cedex 9, France; 4 LUNAM, Université d’Angers, Département de Neurochirurgie, CHU d’Angers, 4 rue Larrey, 49933, Angers, Cedex 9, France; 5 LUNAM, Université d’Angers, INSERM U1066 « Micro- et nano-médecines biomimétiques », bâtiment IRIS 3e étage, CHU d’Angers, 4 rue Larrey, 49933, Angers, Cedex 9, France; 6 LUNAM, Université d’Angers, Laboratoire d’épidémiologie, ergonomie et santé au travail, EA 4626 F-49000, Angers, France; University G. d'Annunzio, ITALY

## Abstract

Many of the brain structures involved in performing real movements also have increased activity during imagined movements or during motor observation, and this could be the neural substrate underlying the effects of motor imagery in motor learning or motor rehabilitation. In the absence of any objective physiological method of measurement, it is currently impossible to be sure that the patient is indeed performing the task as instructed. Eye gaze recording during a motor imagery task could be a possible way to “spy” on the activity an individual is really engaged in. The aim of the present study was to compare the pattern of eye movement metrics during motor observation, visual and kinesthetic motor imagery (VI, KI), target fixation, and mental calculation. Twenty-two healthy subjects (16 females and 6 males), were required to perform tests in five conditions using imagery in the Box and Block Test tasks following the procedure described by Liepert et al. Eye movements were analysed by a non-invasive oculometric measure (SMI RED250 system). Two parameters describing gaze pattern were calculated: the index of ocular mobility (saccade duration over saccade + fixation duration) and the number of midline crossings (i.e. the number of times the subjects gaze crossed the midline of the screen when performing the different tasks). Both parameters were significantly different between visual imagery and kinesthesic imagery, visual imagery and mental calculation, and visual imagery and target fixation. For the first time we were able to show that eye movement patterns are different during VI and KI tasks. Our results suggest gaze metric parameters could be used as an objective unobtrusive approach to assess engagement in a motor imagery task. Further studies should define how oculomotor parameters could be used as an indicator of the rehabilitation task a patient is engaged in.

## Introduction

Motor imagery (MI) consists of imagining the execution of a simple or complex movement that is not accompanied by bodily movement. Thus, MI is an active cognitive process in which the action representation is internally reproduced within the working memory, without overt execution [1] [2] but in a way that is similar to when the subject actually performs the movement [3]. At least two different modalities of imagery can be described [4] [5]: 1) the subject produces a visual representation of the movement; this is also known as visual imagery (VI); and 2) the subject carries out a mental simulation of the movement, which is associated with kinesthetic sensations. This requires imagining “feeling” the movement, and is known as kinesthetic imagery (KI). Motor observation (MO) consists of observing an action realized by other subject. At least, two different modalities of MO can be described [6]: 1) a passive observation paradigm where participants observe the movement without any overt aim; and 2) an active observation paradigm where participants observe the movement with the intent to reproduce it.

A growing number of neuroimaging studies have demonstrated that many of the brain structures involved in performing real movements also show increased activity during imagined movements [7] or during motor observation [8]. Although the extent of the shared neural overlap between these motor conditions is the subject of current debate [9], action execution, MO and MI share similar neural networks and mechanisms [10] [11] at least in part, and this might be the neural substrate underlying the effects of MI in motor learning and motor rehabilitation.

One of the applications of MI ability is the use of MI either as a training process in sports [12], or as a rehabilitation process [13] [14] [15] [16]. Sports literature indicates that MI in combination with physical exercise improves motor function more effectively than either MI or exercise [17]. MI has also been studied as a rehabilitation procedure in patients unable to perform active movements [18] [19] [20] or, when possible, in association with performing actual movements. A systematic review concluded that there is evidence that MI combined with physical therapy has additive effects on motor recovery after a stroke [13], while MI alone does not necessarily result in greater performance [21] [22].

Despite these findings, there are a number of differences in the available neuroimagery literature concerning the neural network involved in MI [13] [22] which appears to vary between VI and KI tasks, and also concerning the effects of either type of MI on motor rehabilitation. Some of these differences may be due to methodological limitations, since monitoring of the type of activity a subject is really engaged in when instructed to imagine a movement is difficult. Such monitoring is most often lacking or limited to verbal feedback from the subject on the task, reporting of the vividness of the mental representation of movement [23] or mental chronometry tasks. The mental chronometry method provides information about the temporal congruence between real and imagined movements. This method has been proven to give reliable and replicable results in healthy subjects and stroke patients (for a review see Malouin et al. [24]). Mental chronometry requires the subject to imagine the movement and give “go” and “stop” signals allowing measurement of the time required for the mental procedure. However, in the absence of any objective physiological measurements it remains impossible to be sure that the patient is indeed performing the task as instructed. The combination of existing measurement methods such as mental chronometry and task-related electrophysiological measurements would be necessary to assess objectively the real engagement of the subject in the MI task, which is a covert cognitive task.

Measurement of eye movement during a MI task could be a possible way of “spying” on the activity the patient is really engaged in. Indeed, eye movements have been studied in a variety of motor tasks, including movement performance, MO and MI [11]. The gaze metrics commonly measured in this experimental approach are fixations, the brief periods of time (typically greater than 100ms) when the eyes are stable and consciously focused on a visual cue [25]. Fixation can be described both by spatial and temporal parameters (describing how long the subject fixes a given point in space). McCormick and al. reported significantly longer fixation duration in MO compared to MI tasks [26], and that fixation duration was significantly influenced by target size and task complexity in MO tasks and action execution tasks, but not in MI tasks [25]. Another possible parameter is the measurement of saccades, which are the rapid eye movements between one fixation and another. The saccade time has been studied across a variety of cognitive tasks [27] [28] [29]. Similarities in eye movements across motor conditions has revealed that these tasks share a common representation of movement that also governs the eye movement pattern [30] [11]. Comparison of eye movement parameters between conditions might therefore be a possible measure to monitor whether a subject is sufficiently engaged in a MI task. In other words, determining the degree of similarity between the eye movement pattern during MI and during other cognitive processes might be a monitoring procedure to detect whether the subject is indeed imagining the movement. The aim of the present study was to compare the eye movement patterns during motor observation, visual and kinesthetic motor imagery, target fixation, and mental calculation.

## Materials and Methods

### Subjects

Twenty-two healthy right-handed volunteers (16 females and 6 males) (mean age = 25.09 years, SD = 7.05, range = 20–53 years), with normal or corrected to normal vision, participated in the study after giving informed written consent. Inclusion criteria were: age above 18 years, volunteer status, with no limitation of mobility or movement disorder, and right handed as assessed by the Edinburgh Handedness Inventory Questionnaire. Approval was granted by the local Ethics Committee (University Hospital of Angers, France).

### Eye tracking system (*SMI* (SensoMotoric Instruments) System)

The SMI RED250 system (RED: Remote Eye-tracking Device) was used in this study. This device provides the point of regard or gaze direction in real time without any physical contact with the person being tested. This dark pupil eye tracking system uses infrared illumination; the eye and face reflect this illumination but the pupil absorbs most infrared light and appears as a high contrast dark ellipse. The images of the eyes are analysed in real time to detect the pupil and the corneal reflection. To determine gaze points, the system measures the vector distance between the center of the pupil and the corneal reflection (the first Purkinje image). The corneal reflection location compensates for small head movements.

The SMI RED250 system has a gaze position accuracy of 0.4° and a spatial resolution of 0.03°. The sampling rate used in this study was 120 Hz.

The RED device was combined with an external 22” TFT monitor (screen resolution of 1680x1050 pixels) and a laptop that monitored the camera equipment and processes using the SMI iView X^™^ software. The gaze tracking experiment with the presentation of several visual stimuli was created and controlled using SMI Experiment Center^™^ software. The gaze tracking data were analysed using SMI BeGaze^™^ software.

A calibration procedure is needed before using eye tracker. During the calibration session, iView X^™^ software establishes a relationship between the position of the eye in the field of the camera and a point on the stimulus monitor. A calibration session was carried out before each experiment. We used a 9 point calibration, which provided a good compromise between time and accuracy of the calibration. The observer had to fix the calibration points (known locations on the monitor) while the position of the subject’s gaze was recorded by SMI iView X^™^ software in order to create a mapping function relating the positions of the eye to points in the calibration zone.

### Experimental Procedure

The eye-tracking session was performed for each subject in five conditions:

- two motor imagery tasks: visual imagery (VI), kinesthetic imagery (KI),
- one motor observation (MO) task: visual observation of the movement video,
- two control conditions: observation of a fixed image (Fix) and mental calculation (MC).

**Fig 1:**
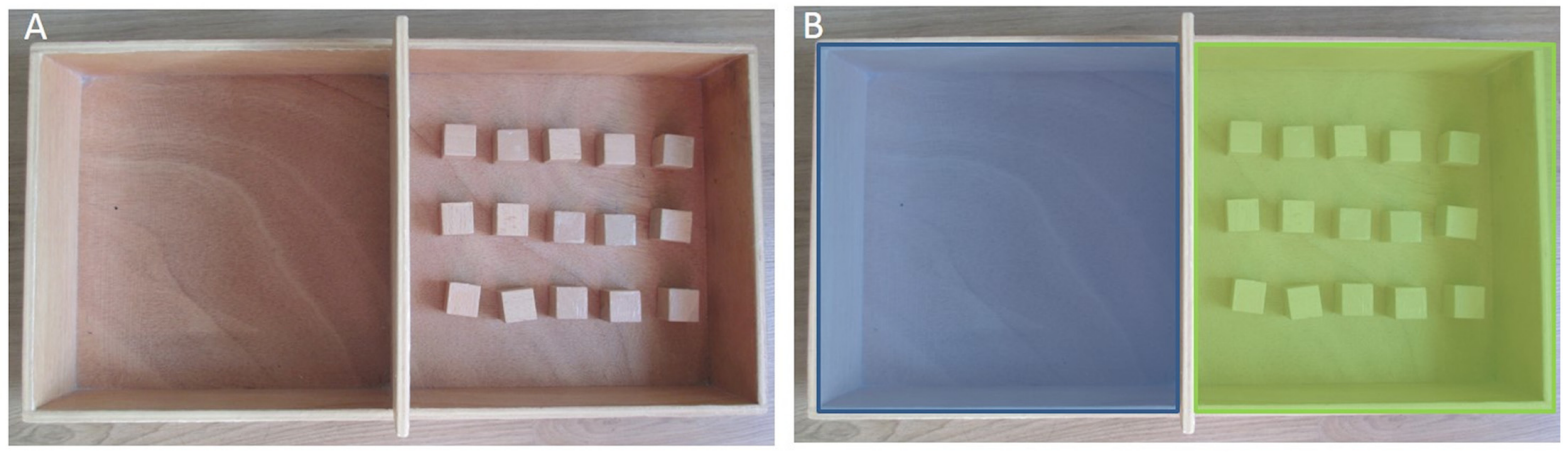
A. Photograph of the Box and Block Test (BBT) used for MC, Fix, KI, and VI conditions, with 15 blocks in the right hand compartment; B. Illustration of the placement of the two Regions of Interest (ROIs) used for counting the number midline crossings.

Before the eye-tracking session, all participants completed the French Movement Imagery Questionnaire-Revised Second version (MIQR-S) as described in Loison et al. [31] in order to assess the imagery ability of each subject. Then they executed the Box and Block Test (BBT) following the procedure described by Liepert et al. [32]. Each subject was comfortably seated at a table on which the BBT was installed with 15 blocks in the right hand compartment (Fig 1A). We ensured that the subject could see both compartments and the cubes inside. He/She received the instruction to pick up each block one by one with the right hand and move it to the left hand side of the box as quickly as possible, with advice to start with the first row and finish with the last row. The examiner demonstrated the movement. This condition served as a procedure enabling the subject to learn the movement in order to imagine it.

For the eye-tracking session, the subject was placed in a comfortable sitting position in front of the RED module and the stimulus monitor. The illumination of the testing room was kept constant throughout the testing session. The subject was requested to limit both head movements and speech during the eye-tracking session. The SMI iView XTM software guided the optimal subject placement for good eye tracking, with two white dots representing the eyes and arrows to indicate the optimal position.

The viewing distance was around 70 cm and the visual angle was 45 pixels per degree.

The order for the MI tasks, MC and Fix conditions was selected randomly. The MO task was presented last, to avoid recalling of the observation task when performing other tasks notably MI tasks. Before each task, the subject read the instructions displayed on the screen and pressed the keyboard space bar in order to start the task.

#### Motor imagery tasks

We used imagination of the Box and Block Test (BBT) tasks following the procedure described by Liepert et al. [32] [33], with two MI conditions (KI or VI). The subjects were shown a photograph of the BBT with the 15 blocks in the right hand compartment. The color of the BBT materials was neutral (wood, see Fig 1A), as color has been reported to increase fixation duration [34]. The same BBT equipment was used to create the photograph and to perform real execution of the movement. For the VI task, participants were instructed to “*Attempt to* *see* *yourself making with your right hand the BBT just performed with as clear and vivid a visual image as possible*”. For the KI task, they were instructed to “*Attempt to* *feel* *yourself making with your right hand the BBT just performed*, *without actually doing it*”. These instructions for VI and KI conditions were derived from the French version of the MIQ-RS [31]. Subsequently no instruction on the perspective (First or third person) used to perform MI tasks was explicitly given to the participants.

#### Motor observation task

**Fig 2:**
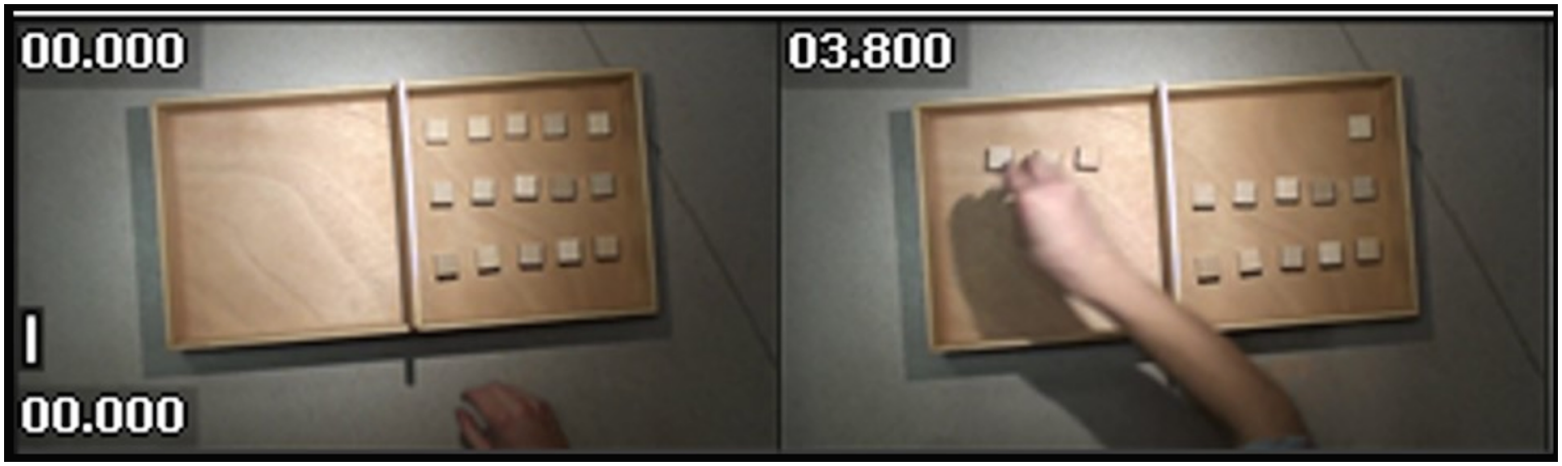
Two snapshot of video used for MO task, showing the arm and the hand of the model.

During the MO task, each participant observed a video showing the execution of the BBT by a third person, moving the 15 blocks from the right compartment to the left with the right hand. Subjects received the instruction to “*observe the video*”. Only the arm and the hand of the actor were visible (for an example see the snapshot of the screen provided in Fig 2). No further instruction and specifically no instruction to imitate the movement was given and the MO task was the last task of the session in order to avoid possible influence of this video on the strategy used by the participants during MI tasks.

To maintain ecological validity the video was a color video of a model performing the BBT. However, the materials of the box and blocks were neutral, as in the photo (Figs 1 and 2). A video of a model has already been used as an observation task in eye gaze studies [26].

#### Control tasks

Two control conditions were included in the experiment to ensure that the gaze metrics were task-related. The first control task was a fixation task (Fix). The participant observed the photo of the BBT used in the MI tasks for 15 seconds, without any specific instruction other than to watch the screen. The second control task was a mental calculation (MC) task during which the participant was instructed to count mentally forwards from 150 in steps of 3 to the first number above 175, and to give the number obtained while observing the same photo of the BBT.

#### Data processing

The projected Point Of Regard (POR), i.e. the (x,y) coordinates of the user’s gaze on the stimulus image or video, was estimated by SMI BeGazeTM software on the basis of gaze tracking data. The software has a built-in saccade, fixation and blink detector. The software considered an out of track period as a blink; this state, in which the eye tracking system cannot determine where the observer is looking, can indeed occur during a blink but also when the observer’s head movement exceeds the tracking capabilities of the system setup. Fixation was defined as a stable gaze position (i.e. within 2.23° visual angle) that was maintained for at least 80ms. Saccades were defined as rapid eye movements repositioning the fovea between two successive fixation periods.

The events (fixations, saccades and blinks) were exported to a tab-delimited table in text format to be analysed with Microsoft Excel^®^ software. The table included the chronological list of events for each event:

- timestamp of the event (start time and end time in microseconds),
- eye position on x, and eye position on y,
- type of event detected (fixation, saccade or blink),
- task and associated stimuli (still images for control conditions and MI tasks, video for MO)

From this information, the total duration of saccades and the total duration of fixations were calculated for each task. We then defined a ratio considered to be the ocular mobility index (OMI) (Eq 1):

$$OMI=100*\frac{Saccade\;duration}{Fixation\;duration+Saccade\;duration}$$ (1)

In order to characterize further the gaze pattern, we used a Region of Interest (ROI) approach to determine whether there was a difference in the number of times the subjects gaze crossed the midline of the BBT when performing the different tasks. This parameter indicates whether two successive eye fixations were or not located in the same half of the BBT. The screen was divided in two ROIs (Fig 1B) placed on each side of the midline of the BBT. The ROI of each fixation period was defined. We then calculated the number of midline crossings. Midline crossing was defined as the fact that the ROIs of two successive fixation periods were situated alternatively on the right and left side (or inversely).

### Mental Chronometry data

For each MI condition (VI or KI), the difference (time lag) between MI duration (recorded by timestamp of the event (KI or VI)) and the real BBT task duration (performed at the beginning of the experiments) was taken as an absolute error and was chosen as a measure of MI time congruence which is frequently used as a marker of MI ability [23] [33].

### Statistical analysis

Analyses were performed using version 6.01 GraphPad Prism for Windows (GraphPad Software, San Diego California USA, www.graphpad.com).

Estimates of the eye-tracking measures are usually quite noisy and are highly sensible to the negative influence of possible outliers. Before analyzing the eye gaze data recorded and to assess the quality of these data, we calculated for each condition and for each participant the percentage of errors/blinks. Then we performed a statistical approach to identify and remove potential outliers using the ROUT method (Q coefficient = 1%) [35]) available on GraphPad Prism 6.01.

After assessing the normality of distributions using Skewness and Kurtosis tests, we carried out a one-factor analysis of variance (one-way ANOVA) with repeated measures for conditions (5 levels; MC, Fix, KI, VI and MO) as within-subject factors, followed by a post- hoc Holm-Sidak’s multiple comparison test to identify the differences between OMI measured in KI and/or VI conditions and in other conditions (MC, Fix and MO). The significance level was set at 5%, adjusted for multiple comparisons. The same analysis was applied to the number of midline crossings.

MIQ-RS results and time lag measures for VI and KI were compared using a paired t test.

## Results

For our sample (22 subjects), the mean value of the percentage of errors/blinks was 8.718 (SD = 5.671) for the MC task, 2.958 (SD = 3.203) for the Fix condition, 5.087 (SD = 4.498) for the KI task, 5.373 (SD = 4.453) for the VI task, and 1.875 (SD = 3.233) for the MO. The ROUT method detected and removed four subjects. Eighteen subjects were subsequently analysed.

### *MIQ-RS* data

The mean value of the MIQ-RS was 5.728 (SD = 0.7598) for the VI tasks, and 4.741 (SD = 0.8555) for the KI tasks.

The paired t test revealed significantly difference between MIQ-RS-VI and MIQ-RS-KI (mean difference = - 0.9864; t = 4.2940; adjusted p value = 0.0004).

### Time lag measures in *KI* and *VI* conditions

The mean value of the time lag was 8.644 seconds (SD = 5.927) for the KI task, and 3.930 seconds (SD = 3.848) for the VI task.

The paired t test revealed significantly difference between KI time lag and VI time lag (mean difference = 4.715 seconds; t = 2.764; adjusted p value = 0.0133).

### Ocular mobility index (Fig 3)

**Fig 3:**
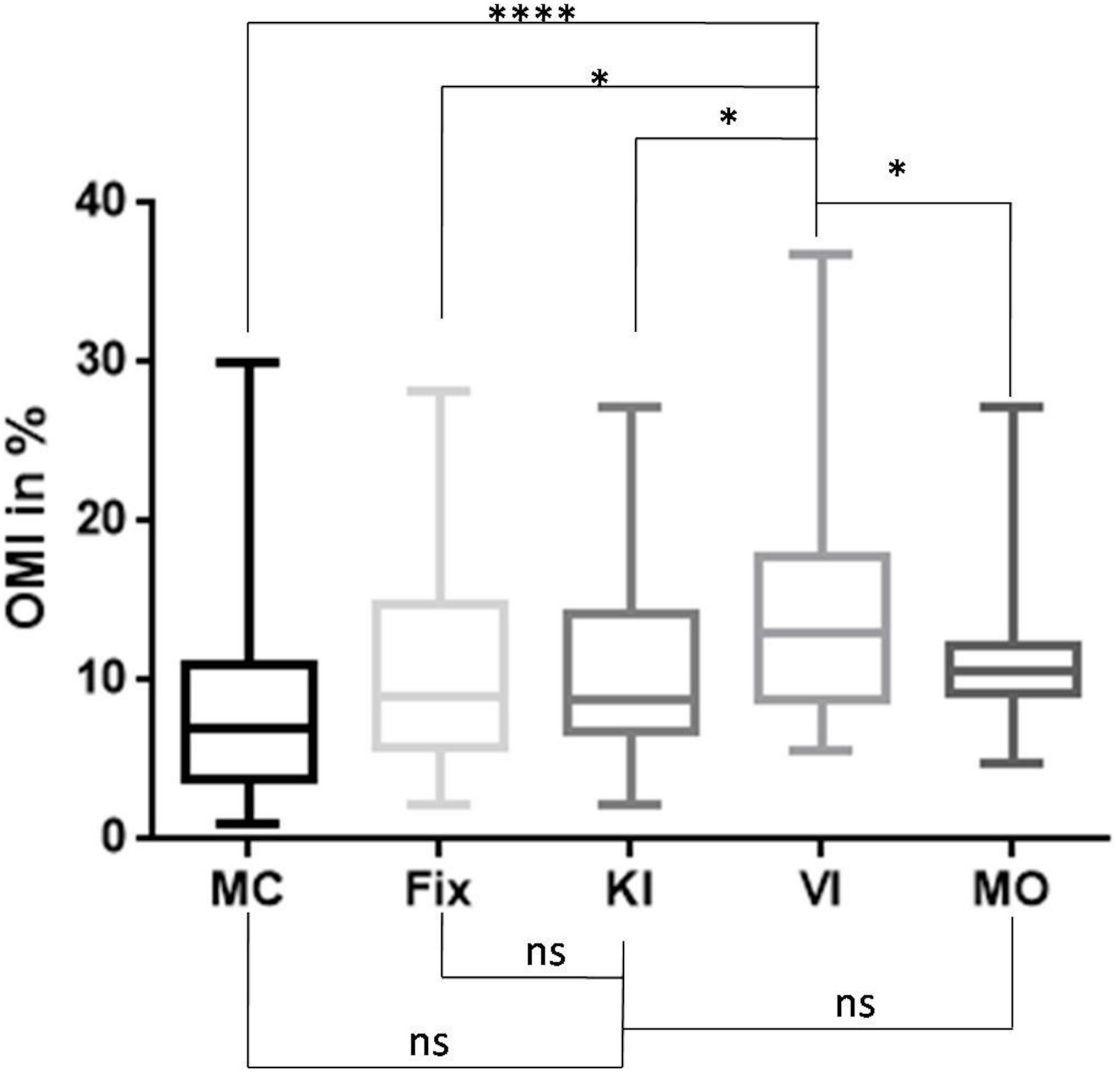
Box plot of the Ocular Mobility Index (OMI = saccade duration/(fixation duration + saccade duration). MC = Mental Calculation, Fix = Fixation task, KI = Kinesthetic motor Imagery, VI = Visual motor Imagery, MO = Motor Observation. The whiskers are drawn down to the 10th percentile and up to the 90th. **** p < 0.0001, *p<0.0500.

The mean value of the OMI was 7.745 (SD = 6.588) for the MC task, 10.04 (SD = 7.578) for the Fix condition, 9.843 (SD = 5.210) for the KI task, 14.16 (SD = 7.942) for the VI task, and 11.22 (SD = 4.551) for the MO.

The one-factor analysis of variance (ANOVA) with repeated measures for the five conditions (MC, Fix, KI, VI and MO) as within-subject factors showed means were significantly different (F = 5.791, p = 0.0005).

The post-hoc test revealed that OMI was significantly different between VI and KI (mean difference = 4.315; SE of dif = 1.380; t = 3.125; adjusted p value = 0.0156, DF = 68); VI and MC (mean difference = - 6.413; SE of dif = 1.380; t = 4.645; adjusted p value = 0.0001, DF = 68), and VI and Fix (mean difference = 4.119; SE of dif = 1.380; t = 2.984; adjusted p value = 0.0196, DF = 68), but not between VI and MO (mean difference = 2.943; SE of dif = 1.380; t = 2.131; adjusted p value = 0.1388, DF = 68). The Holm-Sidak’s multiple comparison test for comparison of OMI between the KI condition and the other conditions did not reveal any significant differences for MC (mean difference = - 2.098; SE of dif = 1.380; t = 1.520; adjusted p value = 0.3487, DF = 68), Fix (mean difference = -0.1958; SE of dif = 1.380; t = 0.1418; adjusted p value = 0.8876, DF = 68), or MO (mean difference = - 1.372; SE of dif = 1.380; t = 0.9939; adjusted p value = 0.5427, DF = 68).

### Region of interest (*ROI*)–based approach, number of midline crossings (Fig 4)

**Fig 4:**
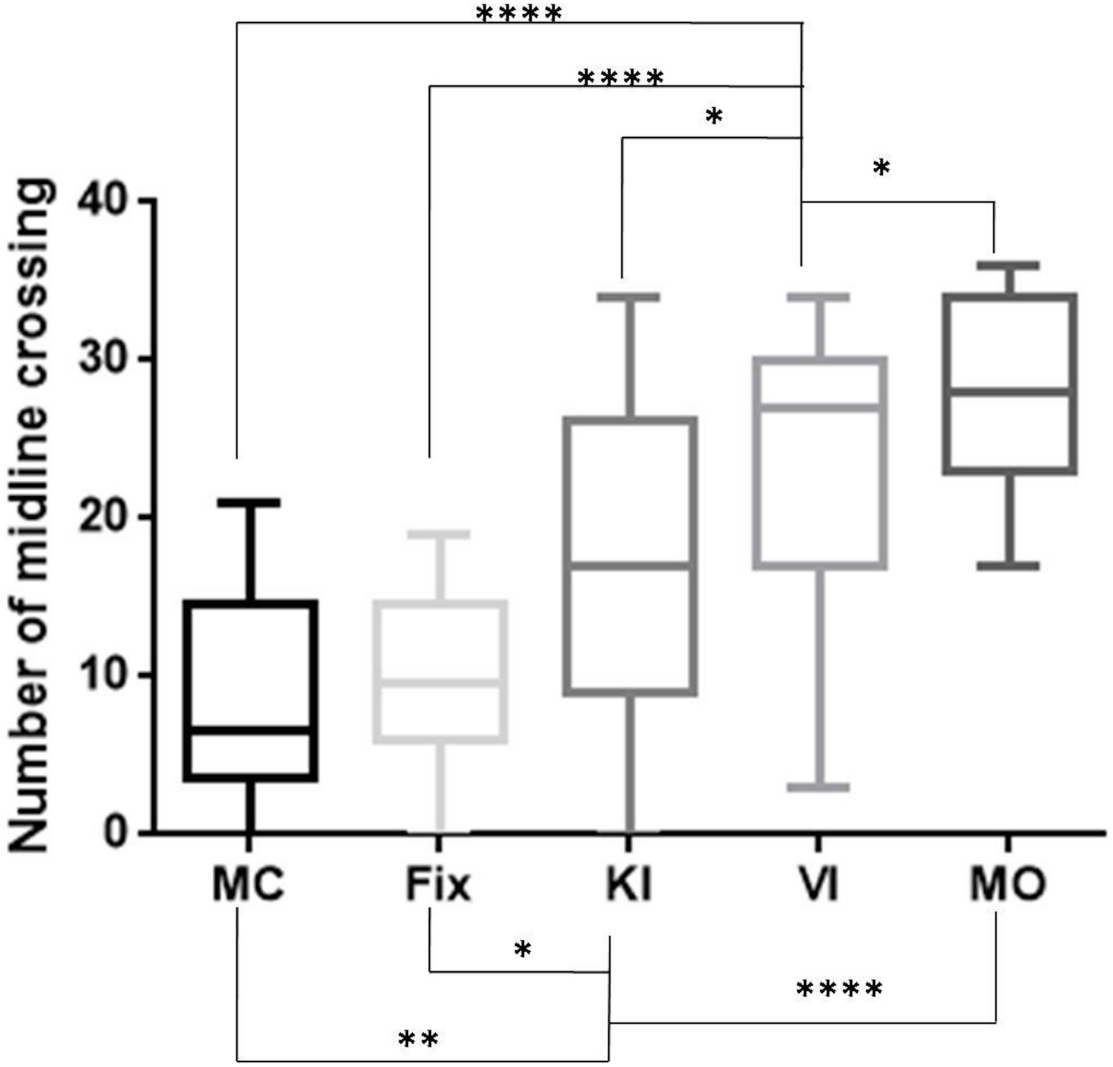
Box plot of the number of midline crossings calculated with the ROIs of the BBT. MC = Mental Calculation, Fix = Fixation task, KI = Kinesthetic motor Imagery, VI = Visual motor Imagery, MO = Motor Observation. The whiskers are drawn down to the 10th percentile and up to the 90th. **** p < 0.0001, *p<0.0500.

When considering the ROI of successive fixation periods, the mean number of midline crossings was 8.444 (SD = 6.252) for the MC task, 9.944 (SD = 5.713) for the Fix condition, 16.83 (SD = 10.29) for the KI task, 23.00 (SD = 9.293) for the VI task, and 28.28 (SD = 5.529) for the MO.

The one-factor analysis of variance (ANOVA) with repeated measures for the five conditions (MC, Fix, KI, VI and MO) as within-subject factors showed means were significantly different (F = 23.77, p < 0.0001).

The post-hoc test (Holm-Sidak’s multiple comparison test) revealed that midline crossings were significantly different between VI and KI (mean difference = 6.167; SE of dif = 2.451; t = 2.516; adjusted p value = 0.0283, DF = 68), VI and MC (mean difference = - 14.560; SE of dif = 2.451; t = 5.938; adjusted p value < 0.0001, DF = 68), VI and Fix (mean difference = 13.06; SE of dif = 2.451; t = 5.326; adjusted p value < 0.0001, DF = 68), VI and MO (mean difference = - 5.278; SE of dif = 2.451; t = 2.153; adjusted p value = 0.0349, DF = 68), KI and MC (mean difference = - 8.389; SE of dif = 2.451; t = 3.422; adjusted p value = 0.0042, DF = 68), KI and Fix (mean difference = 6.889; SE of dif = 2.451; t = 2.810; adjusted p value = 0.0193, DF = 68), and KI and MO (mean difference = - 11.44; SE of dif = 2.451; t = 4.668; adjusted p value < 0.0001, DF = 68).

## Discussion

Our results showed that the metrics of gaze behavior differed in MI tasks, MO and control tasks and suggest that gaze metric parameters could be used as an objective unobtrusive approach to assess engagement in a motor imagery task, as suggested before [36]. We used a non-invasive oculometric system and, due to the inconspicuous RED device, subjects quickly forgot the gaze acquisition system during the test, increasing the reliability of the results. For the first time we demonstrated that eye movement patterns differ between VI and KI showing that these two MI modalities share different cognitive processes. The differences found in the time lag between VI and KI could also support this view.

Previous studies [26] [37] have already established some eye movement congruency between MI and MO tasks, notably by studying the saccadic eye movements as in our study. Previous studies have focused on fixation rather than saccades. As MI tasks and eye movement (specially saccades) share common neural networks, it seemed relevant to include the parameters describing saccades during different tasks, and this led us to the definition of the OMI, corresponding to the saccade duration over saccade + fixation duration ratio.

Indeed, some of the brain areas activated in MI tasks are also involved in control of eye movements [11]. At least four frontal regions participate in eye movements: the frontal eye field (FEF), which participates in voluntary saccades, the supplementary eye field (SEF) located in the rostral supplementary motor area (SMA), the dorsolateral prefrontal cortex (DLPFC) [38] and the pre-SEF. The ‘cingulate eye field’ (CEF) in the cingulate cortex, located at the limit between Brodmann areas 23 and 24, is involved in intentional saccade control to act in forthcoming motor behavior [39]. In the parietal lobe, the posterior parietal cortex (PPC) is involved in the control of saccades and attention, and the parietal eye field (PEF) is involved in triggering a reflex saccade.

However, OMI could appear as a rough indicator of gaze behavior because this index focused on ocular saccades and says nothing about the actual pattern of eye movements during the different tasks. Therefore we used a ROI approach which allowed further study of the gaze pattern by calculating the number of midline crossings. This simple parameter appears well adapted to the BBT which requires moving the blocks from one side of the box to the other when performing real movement and one side of the screen to the other when performing visual imagery.

Using these two parameters (both the OMI and the number of midline crossings), we were able to show that eye movement patterns are different during MI, MO and control tasks (MC and Fix). A more in-depth analyze of our results showed differences in eye movement pattern between KI and VI, and this has not, to our knowledge, been previously described. KI and VI are known to be different cognitive processes and rely on different neuronal networks [4] [7]. Our results support this view, when considering that studying gaze behavior is an indirect approach that offers an objective and dynamic marker of neuronal activity [34].

Neuroimagery studies have shown that MI activates brain areas which are also involved in movement execution, such as the SMA, the superior and inferior parietal lobule, dorsal and ventrolateral pre-motor cortices, pre-frontal areas, inferior frontal gyrus, superior temporal gyrus, primary motor cortex (M1), primary sensory cortex, secondary sensory area, insular cortex, anterior cingulate cortex, superior temporal gyrus, basal ganglia and cerebellum [4] [7] [10] [40]. The modality of MI (KI or VI) seems to influence the consistency of brain activation across MI neuroimagery studies [4] [7]. Activation is greater in occipital areas during VI and in sensori-motor areas during KI, suggesting that VI is a visuo-motor task and KI a sensori-motor task [23]. Thus, the difference in terms of eye movement pattern between VI and KI might indicate either an intrinsic difference between the two tasks, VI being a visuo-motor task and KI a sensori-motor task, or the difficulty encountered by the subject in engaging appropriately in KI compared to VI. Indeed, KI is known to be more difficult than VI as confirmed by the lower KI abilities compared to VI abilities found in our sample and already showed [31] [41].

Behavioral data also show slight, but consistent differences between KI and VI. Our healthy sample population demonstrated lower VI than KI time lag, which is coherent with several previous findings [42]. This difference in time lag found here between KI and VI could be interpreted as an indirect marker of previous findings related to the different cognitive and neural processes activated during these two MI tasks (see above). MI consists in a mental transformation of visual and kinesthetic percept’s [43] and KI, but not VI seems to be influenced by biomechanical constraints and postural manipulation [44] [45] [46]. During VI tasks the participant does not actually encounter the biomechanical constraints of the movements (the participant does not “feel” the movement during VI), and time duration to perform VI is shorter than the time duration to perform KI. Subsequently compared to the actual performance, the time to perform VI is more accurate than the time to perform KI. Thus our eye gaze data and mental chronometry confirm that KI and VI appear as two separate cognitive processes. This warrants further investigation.

Number of midline crossings show a progressive increase of eye mobility from the MC task to MO, VI being the closest to MO. This could be related to the fact that MI and MO are governed by similar activation of the motor system [47]. The difference between VI and MO remains significant for midline crossings but not for the OMI. The explanation of the apparent contradiction in the parameters of MO and VI tasks is not obvious. Methodological considerations such as the small difference in the apparent size of the BBT in MI tasks (40 cm x 20 cm) and in the MO (30 cm x 15 cm) could play a role. Indeed the size of the target may influence saccade duration (saccades do not conform to Fitt’s Law and can decrease in velocity as the distance between two targets decreases). However, the OMI, which used saccade parameters, did not differ between VI and MO. Thus we believe that this possible difference does not dramatically influence our OMI results. Since the result is an absence of difference in OMI despite a difference in target size, this consideration tends to make our result more robust.

The absence of difference in the OMI between these two conditions is in line with previous results. Jacobson [48] and Totten [49] were the first to analyse oculomotor behavior during VI and showed similarities in the production of visual saccades. Hebb [50] later considered VI as a rehearsal of visual perception and hypothesized that VI relied on, and required the production of, ocular movements. Comparison of scanpaths (the resulting series of fixations and saccades) during VI and MO further suggest that internal image representation consists of a sequence of sensory and activities [51] [52] [53]. MI and MO appear to be mediated by the activation of the motor system and organized as motor actions [47]. The oculomotor system would then encode the ocular movements during the perception of a visual scene, and use them to generate the image necessary for VI [54]. The visuo-motor processes activated in MO and VI would therefore be very similar [55] [56], and the ocular movements during VI could be interpreted as an echo of our internal visual representation [37] [57]. In other words VI could be understood as a motor task relying on visual perception in which the subject views his internal representation of movement [26]. This is corroborated by neuroimagery data showing the involvement of the posterior parietal cortex in the visuo-spatial representation of movement [58] [59] [60], and its role in the visual guidance of motor execution [61]. Activation of this area is observed during actual movement execution and also during observation of a third person’s movement [62] [63] and VI [7].

While our results suggest a cognitive proximity between VI and MO as previously described [11], the analysis of the number of midline crossing between these two tasks shows a significant difference in the eye gaze pattern. This result suggests that eye gaze pattern are not similar between MO and VI revealing a difference in terms of cognitive demand between these two tasks, even though the visual component is clearly important in VI task [1]. Here, the lack of neurophysiological data does not allow further discussion and further studies are required. Perspective is an important issue that is often overlooked in motor imagery/motor observation studies. Actions can be imagined from a first person perspective and or a third person perspective, and the agent of the action can be the self or other (in either perspectives). In our study participants could have imagined themself from either a first person or third person perspective and the perspective may not have been consistent between VI and KI. This could interfere with the comparison of VI and KI and deserves further studies.

Our results, and their relation to the underlying neural substrate, may mean that the degree of similarity in oculomotor behavior between MO and VI could indicate whether the subject is fully engaged in the VI task. OMI and midline crossings could be a first quantitative approach of the gaze pattern. Further studies, including more sophisticated modelisation of the gaze pattern are necessary to define other parameters of the gaze metrics such as calculation of the entire gaze trajectory, and measurement of parameters describing the similarity of different trajectories such as the Levenshtein distance [64] which has been already proposed by other authors [65] [66] or MultiMatch approach [67]. Moreover, further analyses, with a strict control of the size of the BBT, specifically dedicated to assess eye pattern parameters during VI and MO tasks are required.

## Conclusion

In conclusion, the metrics of gaze behavior (i.e. OMI and number of midline crossings) differed in MI, MO and control tasks and suggest that these gaze metric parameters could be used as an objective unobtrusive approach to assess engagement in a MI task. For the first time we were able to show that eye movement patterns are different during VI and KI tasks. Our results support this view as VI and KI are known to be different cognitive processes and indicate a potential intrinsic difference between the two tasks: VI being a visuo-motor task and KI, a sensori-motor task. Further studies should define how oculomotor parameters could be used as an indicator of the rehabilitation task a patient is engaged in.

## Supporting Information

S1 Dataset Dataset for each participant (18) concerning MIQ-RS values (behavioral data), number of midline crossing (ROI) and OMI. (XLSX)
